# Optimizing Human Cell-Free System for Efficient Protein Production

**DOI:** 10.4014/jmb.2410.10026

**Published:** 2025-02-25

**Authors:** Abbas Mansour, Kalle Kipper, Arto Pulk

**Affiliations:** Structural Biology Unit, Institute of Technology, University of Tartu, Tartu 50411, Estonia

**Keywords:** Protein, ribosome, translation, in vitro, cell-free

## Abstract

We present a highly efficient human HEK293-based cell-free protein synthesis (CFPS) system capable of producing up to 300 μg/ml reporter protein. One of the limitations of the CFPS systems with respect to protein yield has been the decline of the protein-synthesizing activity of the system upon prolonged incubation. Though factors contributing to this decline in activity have been investigated in yeast, little is known about the factors in mammalian systems. We find that a rapid depletion of the components of the energy-regeneration system is a major factor behind the decreasing protein-synthesis activity in the HEK293-derived system. In addition, we demonstrate that a functional CFPS system can be prepared from other mammalian cell lines as evidenced by our use of a human neuroblastoma SH-SY5Y-derived CFPS system. We also find that exogenous creatine kinase (CK) is dispensable for the functionality of the energy-regeneration system in HEK293 due to the presence of a sufficiently high endogenous CK activity in an HEK293 cell-free extract.

## Introduction

The cell-free protein synthesis (CFPS) techniques use the transcription and translation machinery found in cells to synthesize proteins of interest without the requirement for living cells. *Escherichia coli*-based extracts have been the most common source for CFPS, and many improvements have extended the activity of the system and its productivity [1, 2]. Batch cell-free protein synthesis using optimized *E. coli* extracts has reached milligram quantities of protein per milliliter [3, 4]. Cell-free protein synthesis using the *E. coli* PURE (Protein Synthesis Using Recombinant Elements) system, composed of purified *E. coli* translation factors and elements [1], produced more than 4 mg/ml of protein in dialysis mode [5]. Such a high CFPS quantity has not been reported in eukaryotic systems. In the past decade eukaryotic CFPS systems have become a popular tool and have been widely used to study different aspects of protein synthesis and mRNA biology. Using a CFPS system allows the translation of exogenously added mRNAs in a specific and controlled manner independently of nuclear events [6-8]. The CFPS assay has been used in transcription, splicing, miRNA regulation, translation, synthesizing specific proteins for medical or industrial purposes etc. [6, 7, 9-14]. It offers several advantages over traditional methods of protein synthesis, such as the ability to more effectively control the conditions of protein synthesis and the ability to use non-natural amino acids to create proteins with new properties. The lack of a cell membrane also allows for an easier manipulation of reaction conditions, including the incorporation of labeled amino acids (^13^C, ^15^N) for mass-spectrometry or analyzing the effect of cell-impermeable drugs. By adding micelles, nanodiscs or organelles into the CFPS system, membrane proteins can be studied and purified [15, 16]. Chinese hamster ovary (CHO) cell lysates together with microsomes have been used to produce CFPS derived antibodies [17] or to produce high yields of difficulty-to-express proteins [18]. If the sequence of the seasonal strain of influenza virus hemagglutinin or neuraminidase is known, utilizing the mammalian CFPS system for antigen production offers a rapid method to obtain the antigen for subsequent studies. The yeast *Pichia pastoris* CFPS system was used to produce therapeutically important proteins [19]. Additionally, cell-impermeable drugs can be studied with CFPS assay that is impossible with an *in vivo* assay. The CFPS can be used to redesign the genetic code by utilizing *in vitro*-transcribed tRNAs [20, 21]. Additionally, human cell-derived lysates provide a better environment for the proper folding of human proteins as they facilitate the incorporation of human-specific post-translational modifications [22] which cannot be added by bacterial system. For that reason, the CFPS has been used to produce glycoproteins in a hybridoma based extract [23].

An increasing trend in medicine is to use mRNA-based therapies for treating cancer or for vaccination and a CFPS system can be used to evaluate the therapeutic mRNA expression in a specific cell line or tissue. For instance, the impact of different 5' and 3' untranslated regions (UTRs) on the expression levels of a therapeutic mRNA can be easily assessed in a CFPS system. Algorithms for modelling RNA secondary structure [24, 25] have suggested that UTRs have the potential to engage in intricate RNA base-pairing patterns which may change in response to protein binding and may impact the recruitment of ribosomes [26]. The 5' and 3' UTR lengths vary dramatically among individual genes in higher eukaryotes and can range from a few to thousands of base pairs. In humans the median length of 5'UTR is estimated to be around 218 nucleotides and in yeast 53 nt [26-28]. Therefore, designing the 5' or 3' UTR regions of mRNA can regulate both the quantity of expression and the specificity of expression in particular tissues, ultimately enhancing the efficacy of mRNA-based therapies and enabling targeted gene expression strategies. The most widely used *in vitro* translation system is rabbit reticulocyte lysate (RRL) [29, 30] but it is not very sensitive to cap-dependent translation, the addition of the cap structure to the mRNA has a relatively small effect to the CFPS [31, 32] and it has other caveats [22].

A disadvantage of the CFPS systems as a protein production platform is their higher cost compared to expressing proteins in live cells. To overcome this problem, factors limiting protein yields in the CFPS systems must be identified to enable a cost-efficient protein production. One limiting factor for protein synthesis in eukaryotic CFPS systems is the phosphorylation of translation initiation factor eIF2α at serine 51 upon cellular stress [33]. This limitation can be overcome by the addition of the proteins K3L and GADD34 to the CFPS reaction that reduce the eIF2a phosphorylation at serine 51 [33] or using a designed cell line (HEK293T) that endogenously expresses these proteins [34] or mutating Ser51 to Ala as was done in a mouse embryo fibroblast (MEF) system [31]. This problem is more prevalent in higher eukaryotes as the phosphorylation of eIF2α is mediated by four kinases in mammals each of which responds to distinct forms of environmental stress [35, 36] whereas yeast has only one kinase (Gcn2) [37].

The second major problem is that batch CFPS is only active for approximately 1 h depending on the type of extract (yeast, RRL, HeLa, HEK293, CHO, etc.) used, the additives included, or the techniques applied (*e.g.*, dialysis). As the exact cause of this cessation of protein synthesis after 1 h is unknown then we simply call it time-inhibition. The CFPS time-inhibition beyond 1 h has previously been observed in yeast [38-40], HEK293 [34, 41] or HeLa [6, 22, 42]. Most of the studies on the topic of CFPS time-inhibition are made using the yeast system as it is more affordable and scalable. The dialysis approach to remove accumulated byproducts and feeding the system with energy (ATP, Creatine phosphate) and building blocks (amino acids) has enabled to extend the CFPS activity beyond the 1h cap [40, 43]. However, even when using the CFPS combined with dialysis, the yields of yeast 17 μg/ml (10 h incubation) [40] or in a HeLa system of 50 μg/ml (36 h incubation) [43] have been low. Experiments in the yeast and RRL system indicate that the energy regeneration system is the main cause of the protein synthesis termination as the secondary energy resource creatine phosphate (CrP) and nucleoside triphosphates are rapidly depleted [40, 44, 45]. During the consumption of the energy substrates the accumulation of inorganic phosphate becomes toxic [40, 44].

We are employing the HEK293-based CFPS system to investigate the factors causing the reduction of protein synthesis that occurs during prolonged incubation. We demonstrate that the mammalian CFPS activity can be extended beyond 1 h, and that the main culprit of the cessation of protein synthesis is the energy regeneration system. Using the dialysis approach the HEK293-based CFPS system can produce ~300 μg/ml protein. Also, glucose can serve as an alternative energy source for ATP regeneration, but the protein yield of CFPS with glucose is not as high as it is with CrP. We observe that native creatine kinases are active in the mammalian CFPS extract, eliminating the need for exogenous CK. Additionally, various kinase and proteasome inhibitors are employed to investigate their effects on CFPS.

## Materials and Methods

### Cell Lines and Culture Conditions

HEK293 or HEK293FT (was kind gift from Professor Mart Ustav lab in University of Tartu, Institute of technology) cells grown at 4 × T75 flasks were seeded at density 6,000 cells/cm^2^ on 12 × 15 cm culture dishes and cultivated in Benchstable DMEM + Glutamax (Gibco) supplemented with 10% FBS/ Penicillin (100 u/ml)/Streptomycin (100 μg/ml) (Gibco). Upon reaching a 90% to 100% confluency, the cells were harvested for extract preparation.

SH-SY5Y (ATCC; REF: CRL-2266) cells initial seeding density was 8,000 cells/cm^2^ and cultivated in 10 × 15 cm cell culture dishes DMEM/Ham’s F-12/10% FBS/Penicillin (100 u/ml)/ Streptomycin (100 μg/ml) (Corning, USA) at 37°C/5% CO_2_ under constant humidity. Upon reaching a 90% to 100% confluency, the cells were harvested for extract preparation.

### In Vitro Transcription Reactions

In vitro transcription reactions were performed using PCR products generated with primers encoding a flanking T7 RNA polymerase promoter and a poly-A tail. Transcription reactions were set up in 100 μl, containing 50 mM Tris-HCl pH 7.5, 15 mM MgCl_2_, 2 mM spermidine, 10 mM DTT, 1 u/ml pyrophosphatase (NEB), 4 mM of each NTP, 0.8 u/μl RiboLock RNase Inhibitor (Thermo Fisher Scientific, EO0382, Lithuania), 3.75 μg /ml T7 RNA polymerase and 1 μg PCR-generated DNA. After 3 h incubation at 37°C, buffer was exchange into water by using Zeba 7 kDa MWCO spin columns (Thermo Fisher Scientific, 89883). 0.05 u/μl RQ1 RNase-free DNase I (Promega, USA) and 1 x DNase I reaction buffer (Promega) was added to the reactions, which were incubated at 37°C for 30 min to remove the template DNA. RNeasy Mini Kit (Qiagen, USA) was used to clean up the RNA.

### mRNA Capping and Methylation

Vaccinia capping system (NEB, M2080S) and mRNA Cap 2'-O-Methyltransferase MTase (NEB, M0366S) were used to add the cap1 structure to the mRNA. The capping reaction was set up in 100 μl. First, 60 μg of mRNA in 68 μl water was incubated at 65°C for 5 min and then on ice for 2 min. The 1 x Capping buffer (NEB), 0.5 mM GTP (NEB), 0.2 mM S-adenosyl-L-methionine (NEB), 0.8 u/μl RiboLock RNase Inhibitor (Thermo Fisher Scientific), 0.5 u/μl Vaccinia capping enzyme, and 2.5 u/μl MTase was added. Capping reaction was incubated at 37°C for 90 min. RNeasy Mini Kit (Qiagen) was used to clean up the capped mRNA.

### Preparation of Cell Extracts for CFPS Reactions

Cells were collected by scraping in DMEM media and pelleted in a swing-out rotor (A-4-38) in an Eppendorf 5702 R benchtop centrifuge at 250 ×*g* / 4°C / 5 min. The cell pellets were gently suspended in 3 ml WASH buffer (20 mM HEPES, pH7.5, 150 mM KOAc, 1 mM MgOAc, 2 mM TCEP) and divided into 2 × 2 ml tubes. Cells were pelleted in a fixed-angle rotor in a SIGMA 1-14K benchtop centrifuge at 295 × g /4°C / 2 min. Cell pellets were once more washed. After washing the cell pellets (~ 1 ml volume) were suspended in 1 ml Lysis buffer. 10 ml Lysis buffer contained 20 mM HEPES, pH7.5, 150 mM KOAc, 1 mM MgOAc, 2 mM TCEP, 1 tablet of Complete ULTRA Tablet protease inhibitor (Roche, 05892791001). Suspended cells (~ 2 ml) in Lysis buffer were treated with 300 μg/ml lysolecithin (Sigma-Aldrich, Germany, L4129 in 100% methanol) and incubated on ice for 2 min. Cells were centrifuge at 9,589 ×*g*/4°C for 45 sec in a fixed-angle rotor in a SIGMA 1-14K benchtop centrifuge. The supernatant was discarded and pelleted cells (~ 1 ml) were suspended in 1 ml Lysis buffer supplemented with 50 μM Trolox (Sigma-Aldrich, 238813), 400 u/ml Ribolock RNAse inhibitor (Thermo Fisher Scientific, EO0382), 2.9 μM bestatin (Sigma-Aldrich, B8385), 100 μM PMSF (Sigma-Aldrich, 78830), 1u/ml RQ1 RNase-free DNase I (Promega). Cells were lysed by pushing through a 3-ml syringe with a 26G needle about 8 times, followed by centrifugation at 9,589 ×*g*/4°C/1 min. After centrifugation, the absorbance of supernatant at A_260_ was measured and adjusted to 50 U/ml with Lysis buffer. Extract was aliquoted in 50 μl volumes to avoid multiple freeze-thaw cycles and flash-frozen in liquid nitrogen and stored at -80°C.

### In Vitro Translation Reactions

The optimal concentration of the magnesium and potassium ions was determined to be around 2.5 mM and 160 mM, respectively. HEK293 or SH-SY5Y extracts were first treated with Micrococcal nuclease (NEB, M0247S) to degrade endogenous mRNAs. For this, 50 μl of extract was incubated for 15 min at RT with 0.365 mM CaCl_2_ and 0.2 u/μl MNase. The MNase was inactivated with the addition of 1.45 mM EGTA.

Translation reactions with the MNase treated HEK293- or SH-SY5Y-based CFPS system were set up according to a previously published procedure [8] with modifications. If not stated otherwise the 10 μl of CFPS reaction contained 5 μl HEK293 extract, 39 mM HEPES, pH7.5, 160 mM KOAc, 2.5 mM MgOAc, 0.2 mM spermidine, 0.1 mM putrescine, 2 mM TCEP, 1.3% glycerol, 2 mM creatine phosphate (Sigma-Aldrich, 10621714001), 10 mM amino acids (Promega), 1 mM ATP, 1 mM GTP, 0.8 u/μl RiboLock RNase inhibitor (Thermo Fisher Scientific) and 400 ng IFITM-mRNA.

The HEK293T-based translation reactions were incubated for various times (0 to over-night) min at 30°C and Steady-Glo assay kit (Promega) was used to monitor the luciferase activity. Per 50 μl of Steady-Glo reagent 3 μl of CFPS reaction was used in Nunc 96-well microplate (ThermoFisherScientific, 267350). Glomax luminometer 96 microplate reader (Promega) was used to measure luciferase activity.

For the kinase and protease inhibition experiments, cell-free translation systems were treated with the indicated concentrations of NH125 (Cayman Chemical, 10011250), AT-13148 (Cayman Chemical, 21597, USA), A-484954 (Cayman Chemical, 142557-61-7). 20S proteosome inhibitor Bortezomib was purchased from Sigma-Aldrich (5043140001) and autophagy inhibitor Spautin-1 from Sigma-Aldrich (SML0440).

Since bortezomib and other small-molecule compounds used in this study are not well soluble in aqueous solutions and need to be dissolved in organic solvent e.g. DMSO. Generally, DMSO is used as a solvent for certain compounds in assays, and its concentration should be kept as low as possible to avoid interference with the biological activity. We first tested the effect of DMSO on the CFPS activity. Our results demonstrate that at a DMSO concentration of 0.5%, the CFPS activity is not reduced by more than 15% (Fig. S6). The 5% DMSO decreases CFPS activity 4-fold. In the subsequent small-molecule titration experiments, the concentration of DMSO was therefore adjusted to 0.5%. DMSO titration was conducted using ThermoFisherScientific DMSO, anhydrous (D12345).

Creatine titration was conducted with Sigma-Aldrich (C3630). D-+-glucose was purchased from Sigma-Aldrich (G7528). Myokinase from Sigma-Aldrich (M3003).

In vitro translation reactions with different mRNA’s were set up as described above but with different concentration of mRNA. CrPV-IRES mRNA (1.77 mg of mRNA per 10 ml CFPS reaction), EMCV-IRES mRNA (0.5 mg of mRNA per 10 ml CFPS reaction), and HCV-IRES (1 mg of mRNA per 10 ml CFPS reaction).

### In Vitro Translation Reaction with Dialysis System

The CFPS reaction was set up in 95 μl volume. HEK293 extract was MNase treated as above. The CFPS reaction contained 47.5 μl HEK293 extract, 39 mM HEPES, pH7.5, 160 mM KOAc, 2.5 mM MgOAc, 0.2 mM spermidine, 0.1 mM putrescine, 2 mM TCEP, 1.3% glycerol, 20 mM creatine phosphate (Sigma-Aldrich, 10621714001), 50 mM amino acids (Promega), 1 mM ATP, 1 mM GTP, 0.8 u/μl RiboLock RNase inhibitor (Thermo Fisher Scientific), 3 U/ml pyrophosphates inorganic (NEB, M03615) and 5.65 μg IFITM-mRNA. The CFPS reaction was loaded into the Pierce 96–Well Microdialysis device with a 2K MWCO (A50462), following the company's instruction manual. The membrane was inserted into 2 ml tube that contained 1.5 ml dialysis solution: 40 mM HEPES, pH7.5, 160 mM KOAc, 2.5 mM MgOAc, 0.2 mM spermidine, 0.1 mM putrescine, 1 mM TCEP, 20 mM creatine phosphate (Sigma-Aldrich, 10621714001), 50 mM amino acids (Promega), 1 mM ATP, and 1 mM GTP. The dialysis device opening was covered with Parafilm, and dialysis system was incubated at 30°C at constant shaking (500 rpm) in Biosan TS-100 thermoshaker. After indicated time periods, ~ 6 μl of reaction was withdrawn and 3 μl was used to measure the luciferase activity and rest of the withdrawn sample was flash-frozen in liquid nitrogen and stored at -80°C.

### In Vitro Translation Reactions of the Pellet Fractions

R-dep 50 μl extract was MNase treated by adding 1.5 μl of 25 mM CaCl_2_ and 1 μl of MNase (0.75 U/μl). R-dep was incubated for 15 min at RT. The MNase was inactivated with the addition of 0.6 μl of 51 mM EGTA.

Pellet 1 and Pellet2 MNase treatment was conducted accordingly. To the 50 μl WASH buffer 1.5 μl of 25 mM CaCl_2_ and 1 μl of MNase (0.75 U/μl) was added. The same amount Pellet1 or Pellet2 (A_260_ 37 U/ml) in 10 μl was taken and 10.5 μl of WASH-MNase was added. Pellets were incubated for 15 min at RT. The MNase was inactivated with the addition of 0.6 μl of 51 mM EGTA.

CFPS reactions contained 5 μl R-dep or Pellet1 or Pellet2, 39 mM HEPES, pH7.5, 160 mM KOAc, 2.5 mM MgOAc, 0.2 mM spermidine, 0.1 mM putrescine, 2 mM TCEP, 1.3% glycerol, 2 mM creatine phosphate (Sigma-Aldrich, 10621714001), 10 mM amino acids (Promega), 1 mM ATP, 1 mM GTP, 0.8 u/μl RiboLock RNase inhibitor (Thermo Fisher Scientific) and 200 ng IFITIM-mRNA. The remaining steps were similar to the above-described In vitro translation reaction.

### Immunoblotting

The CFPS batch samples or dialysis samples from different time periods (80 min, 120 min for batch, 120 min, 240 min 300 min, 540 min, 1300 min for dialysis) in 1 × SDS Gel Loading buffer were incubated at 95°C for 5 min and loaded to a 10% SDS-PAGE gel. The commercial luciferase from Photinus pyralis (Sigma-Aldrich, L9420) at indicated amount was loaded to the gel as a concentration reference. The proteins were resolved by electrophoresis at 180 V/50 mA at room temperature. The resolved proteins were transferred onto a 0.45 μm PVDF membrane (Immobilon, Merck) in ice-cold transfer buffer (25 mM Tris, 192 mM glycine; 600 mM MeOH ) at 80V/4°C for 85 min under continuous stirring. The membranes were blocked with 5% milk (nonfat dried Milk, AppliChem, Germany) in Tris Buffered Saline with Tween (TBST) (50 mM Tris-HCl, pH 7.5, 150 mM NaCl, 0.05% (v/v) Tween-20) at room temperature for 1 h with constant shaking. The membranes were incubated in 0.5% milk in TBST for 1 h with the recombinant anti-firefly luciferase antibody (Abcam, ab185924) at a 3:10,000 dilution, followed by three 2-min washes with room-temperature TBST. The antibodies were detected by incubation with an HRP conjugated antibody (Goat Anti-Rabbit IgG (H +L) Peroxidase Conjugated, Pierce 31466; at a 1:10,000 dilution) in 0.5% milk/TBST for 1 h at room temperature, followed by three 2-min washes with room-temperature TBST and incubation in 2 ml ECL (Cytiva, ECL Western Blotting Analysis System) developing solution. The detection and visualization of the protein bands was completed with Hyperfilm ECL (Cytiva). Images were edited using CanvasX Draw software (version 7.0.3 Build 7089): https://www.canvasgfx.com/products/canvas-x-draw

### Nano-LC/MS/MS for Protein Identification

Samples were injected to an Ultimate 3000 RSLCnano system (Dionex) using a C18 trap-column (Dionex) and an in-house packed (3 μm C18 particles, Dr Maisch) analytical 50 cm × 75 μm ID emitter-column (New Objective). Peptides were eluted at 250 nl/min with a 5-35% B 120 min gradient (buffer B: 80% acetonitrile + 0.1%formic acid, buffer A: 0.1% formic acid) to a Q Exactive Plus (Thermo Fisher Scientific) mass spectrometer (MS) using a nano-electrospray source (spray voltage of 2.5 kV). The MS was operated with a top-10 data-dependent acquisition strategy. Briefly, one 350-1,400 m/z MS scan at a resolution setting of R=70,000 at 200 m/z was followed by higher-energy collisional dissociation fragmentation (normalized collision energy of 27) of 10 most intense ions (z: +2 to +6) at R=17,500. MS and MS/MS ion target values were 3e6 and 5e4 with 50 ms injection times. Dynamic exclusion was limited to 40 s.

### LC/MS/MS Raw Data Processing

Mass spectrometric raw files were analyzed the with the MaxQuant software (version 1.6.15.0) [77]. The methionine oxidation was set as variable modifications. Cysteine carbamidomethylation was defined as a fixed modification in both searches. Searches were performed against the UniProt (www.uniprot.org) *Rattus norvegicus* or *Homo sapiens* reference proteome database using the tryptic digestion rule (including cleavages after proline). Transfer of identifications between runs was enabled. iBAQ feature was also enabled, which normalizes protein intensities by the number of theoretically observable peptides and enables rough intra-sample estimation of protein abundance. Peptide-spectrum match and protein false discovery rate (FDR) was kept below 1% using a target-decoy approach. All other parameters were default.

## Results

### HEK293 Based CFPS Assay

**Fig. 1:**
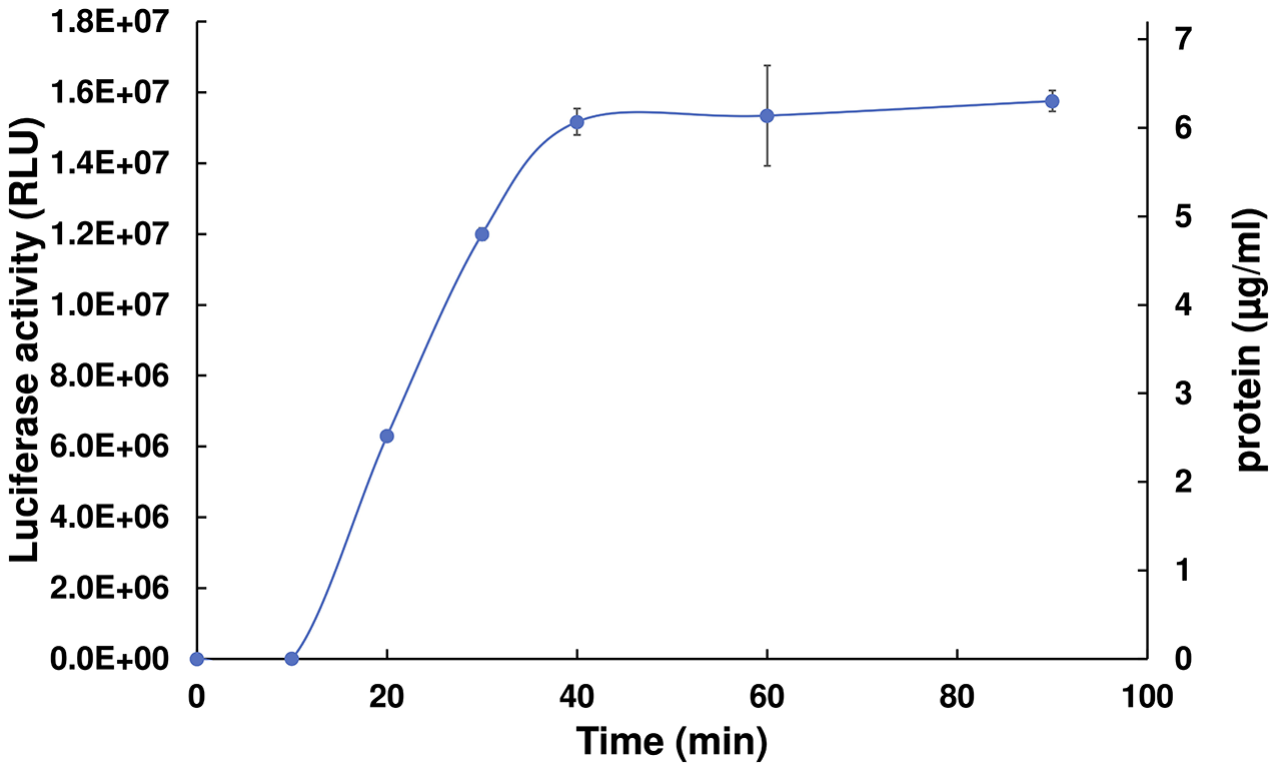
The HEK293-based CFPS activity stopes after a 1-h incubation. The capped-IFITM-Luc mRNA programmed CFPS activity was monitored for 90 min. Luciferase reporter activity is represented as RLU (relative light unit). Luciferase activity reached as high as 16 million RLU after a 40-minute incubation at 30°C. The data represent the average of three experiments with the standard deviation from the mean.

We adapt a previously established HeLa protocol to prepare the HEK293 or HEK293FT cell-free *in vitro* translation extract to improve the protein synthesis extent [8]. Many established human cell line-based CFPS protocols use hypotonic shock to aid the lysis process [6, 8, 22, 34, 41]. However, we are utilizing the lysolecithin approach, where cells are in a physiological buffer [31, 46, 47] and the process itself is faster compared to the hypotonic shock, where a 40-minute incubation is recommended. We employed GADD34 and K3L to enhance CFPS activity [22, 23, 34, 48, 49]. By adding purified GADD34 and K3L to our CFPS system the protein yield is improved by 5- and 3-fold, respectively (Fig. S1A and S1B). We are also adding purified translation factors eIF4E and PABP that improve the translation activity 1.4- and 2-fold, respectively (Fig. S1C and S1D). The other problem with eukaryotic CFPS systems is the diminishing yield after an approximately 1-h incubation period. A few studies on the topic of increasing the CFPS activity beyond 1h have been performed in the yeast or RRL system [40, 44, 45]. We set out to investigate the CFPS time-inhibition in the HEK293 system. When following the established CFPS protocol [8] and adding all the additional proteins (GADD34, K3L, eIF4E, PABP), protein synthesis ceases after a 60-min incubation at 30°C (Fig. 1). To estimate the protein production capacity of the HEK293-based CFPS system on a mass basis, we sought to quantify the amount of luciferase synthesized in units of μg/ml. As our read-out for luciferase is based on the Promega SteadyGlo system, we calculated the amount of luciferase based on the reported conversion factor of 5 × 10^6^ RLU = 0.1 pmol (5 × 10^6^ RLU = 6.1 ng) for firefly luciferase, plotted on the secondary (right) y-axis.

**Fig. 2:**
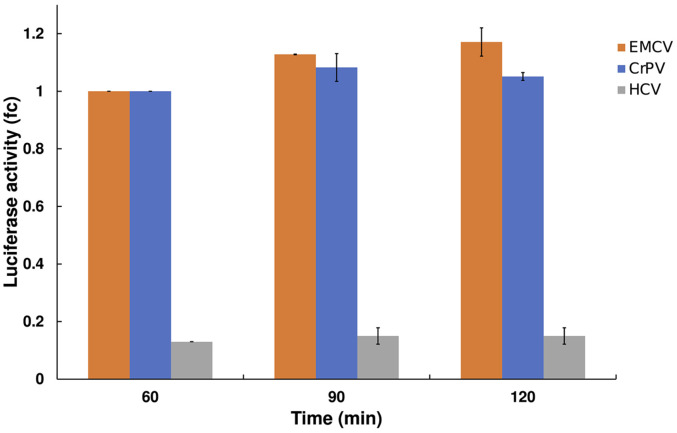
The reduction of CFPS activity is observed after a 1-h incubation with IRES-containing mRNA’s. As with capped IFITIM-Luc mRNA (Fig. 1) we observe similar decrease of CFPS with EMCV-(orange), CrPV-(blue), and HCV- (gray) IRES programmed mRNA’s after 1-h incubation. EMCV- and CrPV-IRES containing mRNA’s have almost similar activities, whereas HCV-IRES mRNA is 8-fold less active. Luciferase activity fold change (fc) is calculated compared to 60 min time point. Only 60- and 120-min time points were monitored for HCV-IRES mRNA. The data represent the average of two experiments with the standard deviation from the mean.

In the majority of the CFPS experiments we are using firefly luciferase as the reporter protein where the luciferase coding sequence in the mRNA is flanked by the 5' and 3' (70 nt poly(A) tail) UTRs of IFITM1 mRNA (IFITIM-Luc). If not indicated otherwise the IFITIM-Luc mRNA is used in these experiments. The mRNA is capped using Vaccinia capping system and the cap1 structure is added by cap 2'-O-Methyltransferase. We have established that the time-inhibition is not dependent on the type of mRNA is used, as the translation of the IRES-containing encephalomyocarditis virus (EMCV), cricket paralysis virus (CrPV) or hepatitis C virus (HCV) mRNAs displays a similar inhibition after 60 min of incubation (Fig. 2). The HCV-IRES containing mRNA showed the lowest yield (8-fold) compared to EMCV and CrPV. As the CrPV-IRES does not require any translation initiation factors [50], this inhibition is likely not related to translation initiation. Furthermore, the inhibition is not dependent on the extract origin as extracts made from the human neuroblastoma cell line SH-SY5Y exhibited a similar time dependent inhibition (Fig. S2). The SH-SY5Y extract was prepared in a similar manner as HEK293, but the difference was that, with the same number of cells, approximately 10-fold less extract was obtained based on the absorbance at 260 nm. This indicates that there are fewer ribosomes in neuroblastoma compared to HEK293.

### Nucleotide Regeneration by Native Creatine Kinases

It is conceivable that the cessation of translation beyond 60 min is caused by either a consumption or inactivation of some component(s) in the extract (ribosomes, translation factors, synthetases etc.) upon prolonged incubation at 30°C. An inactivation may be caused by either a denaturation or a modification (*e.g.*, phosphorylation) of a critical component(s) in the extract.

**Fig. 3:**
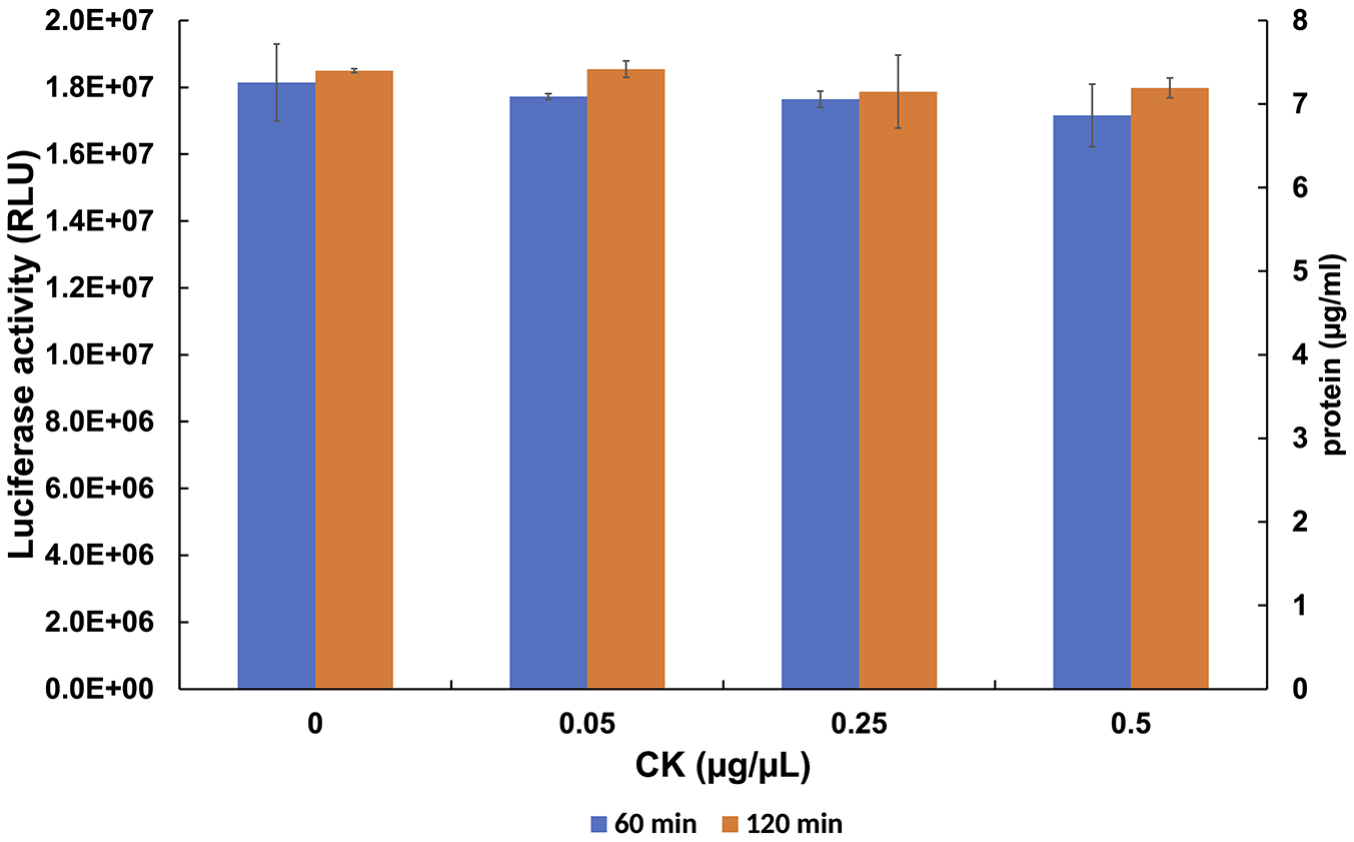
The addition of exogenous creatine kinase (eCK) to the HEK293 CFPS system does not have any beneficial effects. The eCK concentrations 0.05, 0.25, and 0.5 μg/μl were used in CFPS reaction using 2 mM CrP and 10 μM amino acids setup. The CFPS activity was monitored at 60 (blue line) min and 120 min (orange line). The data represent the average of two experiments with the standard deviation from the mean.

The primary source for nucleotide regeneration in CFPS is an externally supplemented creatine phosphate (eCrP) whose high energy phosphate group is transferred to ADP by creatine kinase during ATP regeneration [8, 34]. In the majority of the CFPS protocols, this creatine kinase (eCK) is also supplied externally to the cell-free translation system. To establish an optimal concentration of eCK in the HEK293-derived CFPS, we titrated rabbit creatine kinase up to a concentration of 0.5 μg/μl, including concentrations used in previous studies [8, 34]. Unexpectedly, adding eCK to the CFPS system had no effect on the reporter mRNA translation and the translation was fully active even in the absence of exogenous creatine kinase (Fig. 3). Such a dispensability of eCK suggests that in our system nucleotides are regenerated by endogenous creatine kinases. As an alternative explanation of the dispensability of eCK is that the amount of externally supplemented ATP/GTP was sufficiently high and obviated the need for a creatine kinase-catalyzed nucleotide regeneration. To distinguish between those scenarios, luciferase synthesis at different CrP concentrations was monitored in the absence of exogenous ATP/GTP and creatine kinase. Omission of ATP/GTP in the presence of 2 mM CrP did not decrease the yield of luciferase by more than 2-fold whereas a 133-fold decrease was observed in the absence of CrP (Fig. S3). The substantial residual activity of the HEK293-derived translation system in the absence of added ATP/GTP and creatine kinase thus indicates an abundance of endogenous nucleotide diphosphates and endogenous creatine kinases regenerating ATP from CrP. In agreement with this, a mass-spectrometry analysis of an HEK293 extract depleted of ribosomes revealed the presence of two different creatine kinases: creatine kinase B-type (CKB) and creatine kinase U-type, mitochondrial (CKMT1A). Based on the iBAQ values, CKB is highly abundant in the extract while the level of CKMT1A is approximately 390-fold lower (Table S1). We also note that increasing the eCK concentration did not lead to an increased luciferase synthesis upon extending the incubation time from 60 to 120 min (Fig. 3), indicating that the time-inhibition is not related to creatine kinase. Based on those findings, exogenous creatine kinase was excluded from the subsequent experiments.

**Fig. 4:**
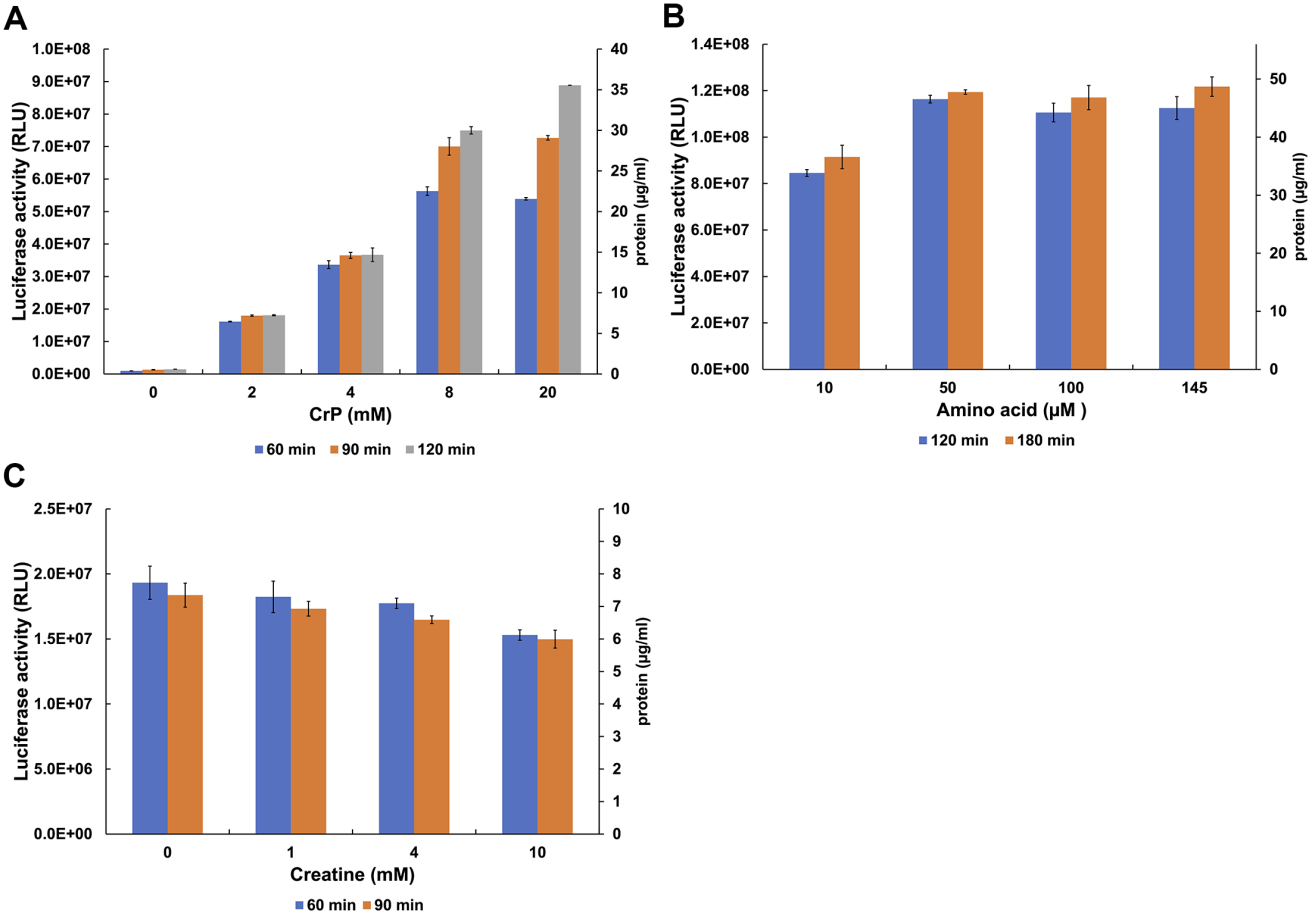
Increasing the concentration of the CrP and amino acids boosts CFPS activity. (**A**) The CrP concentration (0 to 20 mM) was titrated and CFPS activity was monitored at 60 min (blue), 90 min (orange), and 120 min (grey). Increasing the CrP conc. from 2 mM to 20 mM boost the CFPS activity by 5-fold. The CFPS activity is extended by the increased CrP conc. The data represent the average of two experiments with the standard deviation from the mean. (**B**) Amino acid concentration was titrated (10 to 140 μM), while a 15 mM CrP concentration was used throughout all the experiments. The CFPS activity was monitored at 120 min (blue) and 180 min (orange). The data represent the average of two experiments with the standard deviation from the mean. (**C**) Creatine concentration (0 to 10 mM) was titrated against 2 mM creatine phosphate and CFPS activity was monitored at 60 min (blue) and 90 min (orange). The data represent the average of two experiments with the standard deviation from the mean.

As preincubation experiments demonstrated that energy regeneration system is the main culprit of CFPS time-dependent inhibition, CrP concentration was titrated. As the CrP concentration (2 mM) is rather low in the original HeLa protocol [8] on which our CFPS system is based, we titrated the concentrations of CrP and also amino acids in our CFPS system with a view to increasing the protein yield. Compared to the 2 mM CrP in the original protocol, increasing the concentration of CrP to 20 mM led to a 5-fold increase in the CFPS activity upon 120 min incubation (Fig. 4A). Furthermore, at the 20 mM concentration of CrP, the yield of luciferase increased nearly linearly with time between 60 and 120 min (Fig. 4A). At CrP concentrations below 5 mM, this increase disappears (Fig. 4A), indicating that at lower concentrations CrP becomes limiting for translation at least beyond 60 min. Based on those results, CrP was kept at 15 mM in the amino acid titration experiment. In this experiment, we observed a moderate (33%) increase in the CFPS activity upon titrating the amino acid concentration from 10 to 50 μM (Fig. 4B). In contrast to CrP, the yields of luciferase at 60 min and 120 min time points were essentially identical at all amino acid concentrations tested (Fig. 4B and Table S1), indicating that the CFPS activity is not limited by amino acids on this time scale. In a separate experiment, omitting the amino acids from the CFPS altogether caused only a 2-fold decrease in translation activity compared to the activity at 100 μM amino acids (Fig. S3). Apparently, significant amounts of aminoacyl-tRNAs and/or amino acids are present in the HEK293-derived extract.

Since the pool of the added creatine phosphate is used for the regeneration of ATP in the CFPS, creatine (Cr) accumulates in the system upon prolonged incubation. To determine any inhibitory effect of Cr on the CFPS activity, we titrated Cr in the presence of 2 mM CrP up to a Cr/CrP ratio of 5. At this ratio, we observed a 1.3-fold decrease in the CFPS activity (Fig. 4C). As a Cr/CrP ratio of 5 corresponds to an 80% conversion of the initial CrP, this slight 1.3-fold decrease in activity is unlikely to markedly contribute to the time-inhibition.

Aleksashin and colleagues introduced two enzymes, myokinase and diphosphate kinase, into the CFPS system [34]. However, the authors did not specifically investigate the individual effects of these enzymes or the effect of their complete omission on the CFPS. Though our results indicate the presence of sufficient endogenous creatine kinase activity in the HEK293 extract, it is conceivable that our CFPS system could benefit from the inclusion of other nucleotide regenerating enzymes. For instance, as the primary energy-consuming reaction during CFPS is the conversion of ATP to AMP due to the tRNA aminoacylation, it is possible that protein yields in CFPS can be increased by the inclusion of rabbit myokinase. Rabbit myokinase converts AMP to ADP through the transfer of the γ-phosphate from ATP to AMP, with the resulting ADP then converted to ATP by the action of creatine kinase. However, including rabbit myokinase in our CFPS assay did not increase the yield of luciferase or prolong the active phase of protein synthesis (Fig. S4). Although we did not investigate this issue further, we posit that our extract contains active endogenous kinases capable of converting AMP to ADP. This could explain the absence of any observable effect upon the addition of rabbit myokinase. In support of this explanation, mass-spectrometry data of the CFPS extract shows the presence of 3 kinases that are capable of converting AMP to ADP, adenylate kinase isoenzyme 1 (AK1), adenylate kinase 2 (AK2), and adenylate kinase isoenzyme 6 (AK6) (Table S2).

Likewise, the nucleotide diphospate kinase maintains the steady-state level of GTP by transferring the γ-phosphate from ATP to GTP, thus replenishing the GTP pool for several of the GTP-utilizing translation factors [34]. Though we did not conduct any experiments with the diphosphate kinase, a mass-spectrometry analysis revealed the presence of enzymes with similar functions in the HEK293 extract, namely nucleotide diphosphate kinase A (NME1), nucleotide diphosphate kinase B (NME2), and nucleotide diphosphate kinase 7 (NME7)(Table S2).

### The Effect of Proteasome, Autophagy, eEF2K, and AGC-Kinase Inhibitors on CFPS Activity

**Fig. 5:**
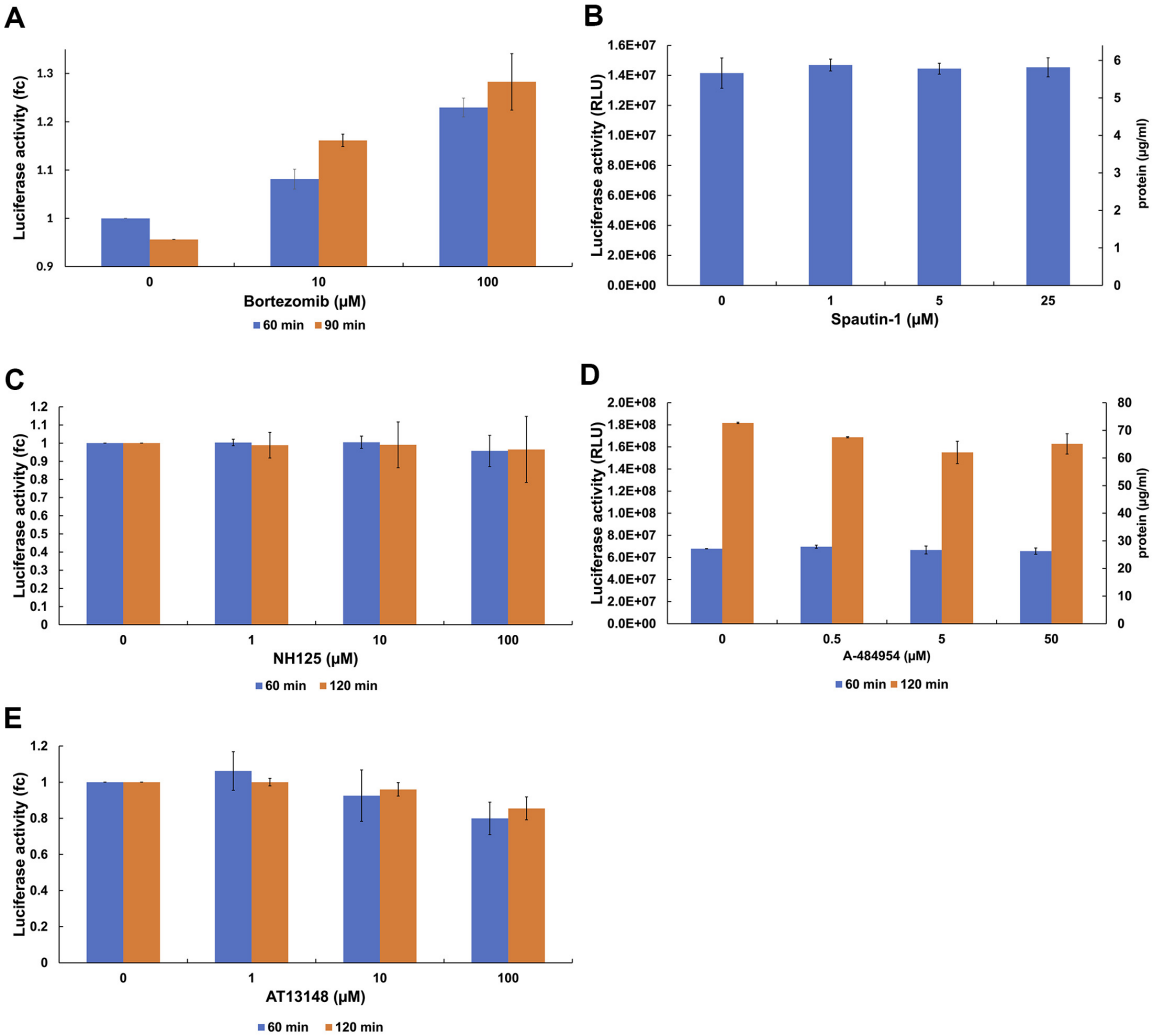
The effect of proteasome, autophagy, eEF2K, and AGC-kinase inhibitors on CFPS activity. (**A**) Proteosome inhibitor bortezomib at conc. 10 μM and 100 μM was used in the CFPS assay and CFPS activity was monitored at 60 min (blue) and 90 min (orange). Fold change (fc) was calculated based on the 0 μM bortezomib concentration. The data represent the average of two experiments with the standard deviation from the mean. (**B**) Autophagy inhibitor spautin-1 at conc. 1 μM, 5 μM, and 25 μM was used in the CFPS assay and CFPS activity was measured after 60 min incubation. No positive or negative effect is observed with spautin-1 addition. (**C**) NH125 (1 to 100 μM) effect on the CFPS was monitored at 60 min (blue) and 120 min (orange). No effect was observed on CFPS activity. (**D**) A-484954 (0.5 to 50 μM) effect on the CFPS was monitored at 60 min (blue) and 180 min (orange). (**E**) AGC kinase inhibitor AT13148 (1 to 100 μM) effect on the CFPS was monitored at 60 min (blue) and 120 min (orange). No effect was observed on CFPS activity. The data represent the average of two experiments with the standard deviation from the mean.

In live cells, ubiquitin-proteosome system uses ATP to unfold the proteins for the degradation of short-lived or damaged proteins [51-54]. It is therefore conceivable that some proteins required for the CFPS are degraded by the proteasome upon prolonged incubation, contributing to the time-inhibition of the CFPS activity. To interrogate the effect of the proteasomal degradation of components of the CFPS system on the CFPS activity, we used the proteasome inhibitor bortezomib (inhibits the chymotrypsin-like activity of the 20S proteasome) in the CFPS system. Inclusion of bortezomib had a moderately positive effect (25%) on the yield of luciferase (Fig. 5A), indicating that some components of the CFPS may be susceptible to degradation upon prolonged incubation of the CFPS system.

Although we note that the respective p-values for bortezomib concentrations of 0 μM and 100 μM are approximately 0.4 and 0.3, indicating that the differences between the mean luciferase activities at 0 μM and 100 μM bortezomib failed to reach statistical significance.

Spautin-1 inhibits the activity of two ubiquitin-specific peptidases, USP10 and USP13, leading to an increase in proteasomal degradation of class III PI3 kinase complexes. These complexes have been shown to regulate autophagy [55, 56]. Autophagy is a process that results in the degradation of long-lived proteins and excess or aberrant organelles, including ribosomal subunits (ribophagy) [54, 57]. Autophagy has been studied in live cells, and it is not clear if it happens in cell-free systems, therefore, we used Spautin-1 inhibitor to test the effect of autophagy of long-lived translation factors or ribosomes in CFPS system. However, on the 1 h time scale spautin-1 did not affect the yield of luciferase in the CFPS within the concentration range tested (Fig. 5B).

The CFPS activity may also be affected by the phosphorylation of elongation factor eEF2 at residue T56 by the corresponding kinase eEF2K which prevents eEF2 from engaging with the ribosome [58]. eEF2 phosphorylation upon *in vitro* translation has recently been reported in HEK293- and HeLa-derived CFPS systems [34]. We therefore tested the effect of eEF2 phosphorylation on the CFPS activity using a small-molecule compound NH125 that affects eEF2-T56 phosphorylation [59-61]. We note that the precise mode of action of NH125 is still debated as it has been reported to either inhibit the eEF2-T56 phosphorylation or conversely, promote it [60]. However, regardless of its mode of action, NH125 did not affect the CFPS activity within the concentration range tested nor did it increase the yield of luciferase when the incubation time was extended from 60 min to 120 min (Fig. 5C).

As the mode of action of NH125 is not completely resolved and it remains to be established whether it is an inhibitor or promoter of the inhibition of eEF2 phosphorylation, we subsequently tested a highly selective small-molecule eEF2K inhibitor A-484954 [60]. We used A-484954 in the concentration range of 0.5 to 50 μM in CFPS assay and incubation times 1 h to 3 h (Fig. 5D). These experiments were conducted with higher CrP (20 mM) and amino acids (50 μM) concentration, compared to other small-molecule inhibitor experiments (2 mM and 10 μM, respectively) and therefor CFPS is not inhibited after 60 min incubation. We did not observe any remarkable effect of A-484954 on the CFPS activity.

We also tested the AGC kinase inhibitor AT13148 which has IC_50_ of 38 nM/402 nM/50 nM, 8 nM, 3 nM, and 6 nM/4 nM for Akt1/2/3, p70S6K, PKA, and ROCKI/II. The mitogen-activated protein kinase (MAPK) and mTOR pathways inhibit eEF2K in response to mitogen and nutrient signals [59, 62-64]. In contrast, AMP kinase-and protein kinase A/Ca^2+^-dependent signaling activates eEF2K in response to starvation, hypoxia, and oxidative stress [59, 65-67]. AT13148 did not have any remarkable effect on CFPS in our experimental setup (Fig. 5E).

### CFPS Energy Source Regeneration

**Fig. 6:**
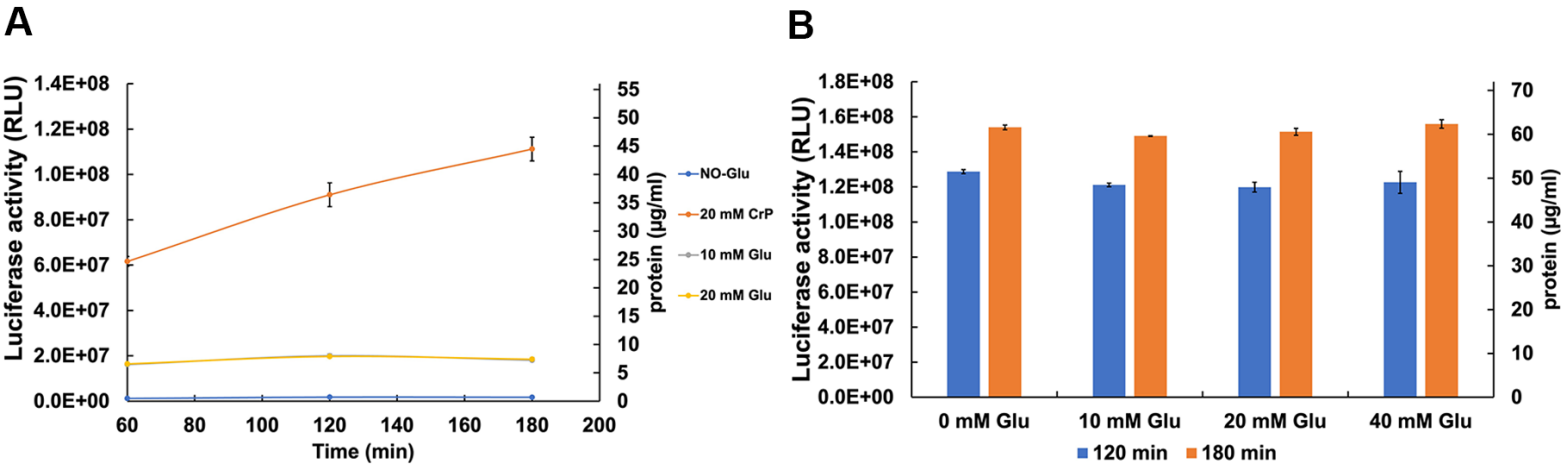
The effect of glucose as the alternative energy source for CFPS. (**A**) Adding glucose (10 mM grey or 20 mM yellow) to the CFPS reaction without supplementing CrP increased the activity by 11-fold compared to glucose omission (blue). The CFPS with CrP without supplementing glucose (20 mM CrP red) was 5.5-fold more efficient compared to 10 mM or 20 mM glucose. The data represent the average of two experiments with the standard deviation from the mean. (**B**) The CFPS activity was measured in the presence of both (CrP and glucose), with variable glucose concentrations (10 mM, 20 mM, and 40 mM) and constant (20 mM) CrP. The addition of the glucose to the dual system did not have any effect on the CFPS. The data represent the average of two experiments with the standard deviation from the mean.

Glucose has been used as a cheap secondary energy source for CFPS in yeast for the replacement of creatine phosphate [39]. It has also been demonstrated that glucose in the RRL-based CFPS system is depleted within a 60 min time period [45]. A yeast CFPS system with glucose produced low levels of protein (3.64 μg/ml) compared to 9 μg/ml with CrP/CrK system but it was 16% more cost effective then CrP/CrK system [39]. Hence, replacing CrP with glucose might be an important biotechnological alternative. In the glucose-based yeast CFPS system, protein production displayed a 2 h lag (probably due to the slow glycolytic ATP regeneration), followed by an active protein production for 2.5 h [39]. We aimed to determine whether glucose has any effect on the human CFPS system. We used two glucose concentrations (10 mM and 20 mM) and for a positive control experiment, 20 mM CrP. The HEK293 extracts exhibit a 5.5-fold higher translational activity with CrP compared to glucose (Fig. 6A). We did not observe any activity difference with increased glucose concentration (10 mM vs. 20 mM), and CFPS reached a plateau in 60 min, whereas CrP activity showed an increasing tendency even at 180 minutes (Fig. 6A). Collectively, those results show that although glucose can serve as a source for ATP regeneration in the HEK293-derived CFPS, the glycolytic ATP regeneration is limited by some factor(s). One limiting factor may be related to the regeneration of NAD^+^ in the absence of mitochondrial respiration.

We also tested the effect of adding glucose in the presence of CrP. In the yeast CFPS system such a dual energy regeneration system severely inhibited protein production [39]. To explore the utility of the Glucose/CrP system in the HEK293-derived CFPS, we titrated glucose in the presence of 20 mM CrP. However, the presence (10 to 40 mM) or absence of glucose in the background of CrP had no effect on protein yield in our system (Fig. 6B).

### Estimating the Protein Yield

Since we use 3 μl out of the 10 μl CFPS sample for luciferase detection, the calculated yields of luciferase after a 180 min incubation were 45 μg/ml at 20 mM CrP and 8.1 μg/ml at 20 mM glucose. In the Western blot assay, we used commercial firefly luciferase witch activity is 10 × 10^10^ U/mg. The luciferase yields based on the commercial luciferase activity would be 66 μg/ml for Glu (20 mM) and 367 μg/ml CrP (20 mM). However, as the commercial luciferase preparation has a significantly reduced activity after a prolonged storage at –80°C and repeated freeze-thaw cycles, we do not consider this estimate entirely reliable. Instead, we used Western blot analysis against the luciferase antibody to estimate Luc concentration. Based on Western blot analysis and band intensity estimation using ImageJ with CFPS results obtained with 2 mM CrP, we estimate that yield with 20 mM CrP would be 80 μg/ml of Luc (Fig. S5). These results demonstrate that our HEK293-based CFPS system is highly active, even in the absence of exogenous CK. Since Aleksashin *et al*. do not report protein yields in their system on a mass basis, a direct comparison of the two systems is not possible [34].

### Enhancing CFPS Activity through the Use of a Dialysis System

**Fig. 7:**
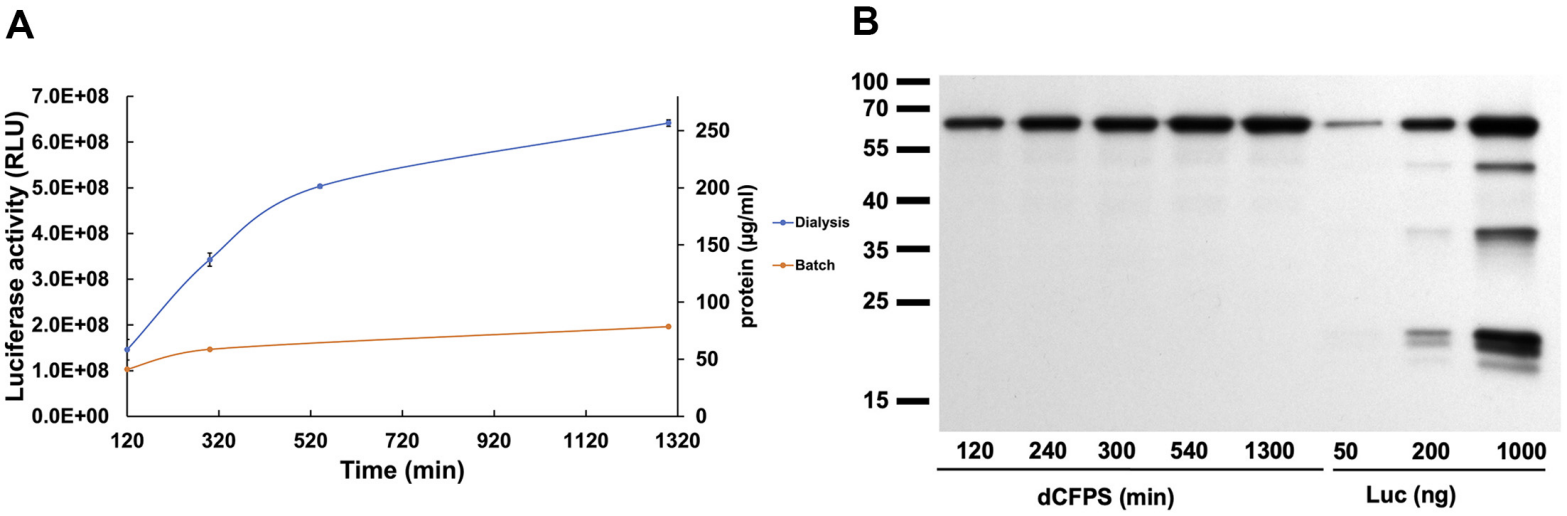
The dialysis approach improves the CFPS activity 4-fold. (**A**) The CFPS activity of 95 μl reaction in the 2K MWCO microdialysis device supplemented with 1.5 ml dialysis buffer containing 40 mM HEPES, pH7.5/160 mM KOAc/ 2.5 mM MgOAc/0.2 mM Spermidine/0.1 mM Putrescine/2 mM TCEP/1 mM ATP/1 mM GTP/20 mM CrP/ 50 μM amino acids. The CFPS activity was monitored overnight. At the same time the batch method (orange) was applied to the same CFPS reaction where 10 μl of CFPS reaction was taken in the beginning to monitor its activity without the dialysis. The data represent the average of two experiments with the standard deviation from the mean. (**B**) At the same time when measuring the CFPS activity of dialyzed CFPS reaction (dCFPS) an aliquot of the reaction was flash frozen in liquid nitrogen and later 1 μl of dCFPS was loaded on protein SDS-gel for western blot analysis. The commercial luciferase with known concentration was used as reference. Left lane shows the protein marker lines in kDa that were added to the image file using CanvasX Draw software otherwise original gels are presented.

The dialysis system has been employed to resupply components for CFPS and to dilute the byproducts in yeast and HeLa systems [40, 43]. We applied the dialysis approach to our HEK293-based CFPS system to determine if a higher protein yield can be achieved. We used a 2K MWCO micro dialysis device with 95 μl of CFPS mixture and 1.5 ml of dialysis buffer containing consumable components (ATP, GTP, CrP, amino acids). The device was incubated at 30°C on the orbital shaker, and time points were taken until overnight. The dialysis system extended CFPS activity beyond 3 h, resulting in a 4-fold increase in protein production (~300 μg/ml) compared to batch-method (80 μg/ml) and based on luciferase activity or Western blot (Fig. 7A and 7B). Completely replacing the dialysis buffer after overnight incubation did not restore CFPS activity. This indicates that some factors have lost their activity and the addition of the amino acids and a fresh energy source does not help to recover the lost activity. Further studies need to be conducted to identify the factors contributing to the inhibition.

## Discussion

For a more informative presentation, we prepared a summarizing table (Table S3) detailing the techniques and compounds described in the manuscript.

We demonstrate that external creatine kinase is not required to regenerate the ATP for CFPS as native CKs are functional. Exclusion of CK was also reported recently in HeLa system, no further protein was synthesized upon addition of the enzyme, further indicating that endogenous CK is sufficient [42]. This knowledge helps to reduce the cost of utilizing the mammalian CFPS assay. The addition of creatine kinase to the mammalian CFPS system may be a remnant of yeast or RRL protocols, given that yeast lacks creatine kinase and it is observed that RRL-based CFPS does not function without CK [45]. Alternatively, the functionality of creatine kinase in the extracts could be influenced by how the extracts are prepared or the specific tissue type. For instance, tissues with large fluctuations in energy metabolism (muscle and nerve) produce large quantities of CK [68]. In skeletal muscle, CrP concentrations may reach 20-35 mM [68], whereas in other tissues such as the brain, smooth muscle, and kidney, it is in the range of 5-10 mM [69]. It would be interesting to use the muscle cell extracts (*e.g.*, SKM-1) for CFPS as muscle cells are specialized for fast regeneration of ATP from CrP. Based on our experiments, it seems that ATP/GTP is quickly consumed in the CFPS system, as adding 1 mM ATP/GTP only produces ~ 1.7 × 10^6^ RLU that equals to 2 ng Luc (Fig. 6). The CK/CrP system is a major source of inorganic phosphate (P_i_) and since P_i_ is required for activation of glycogenolysis and glycolysis, it indirectly regulates these metabolic pathways in muscles [68, 70]. In the yeast CFPS system, adding both (CrP/CK and glucose) inhibited (89%) protein synthesis [39]. In our HEK293 system, we did not observe any inhibition of CFPS with the dual (CrP and Glu) system. However, it did not have any beneficial effects on protein synthesis either. This observation indicates that glycolysis is not very active in the CFPS, although we observe 11-fold boost of CFPS activity upon glucose addition in the CrP omitted system. The glycolytic activity in the HEK293 system may also depend on how much available NAD^+^ or NADP^+^ is in the extract or on the fate of the produced pyruvate etc. Glucose removal in RRL-based system had no apparent effect on steady state ATP and GTP levels, confirming that the CrP/CK system is the primary and essential energy source, but interestingly glucose reduction lowered protein synthesis by about 30% [45]. Glycolysis in RRL appears to contribute to protein translation, either through NADH regeneration or via production of intermediate metabolites that may indirectly benefit translation [45]. In our HEK293-based CFPS system we did not detect any reduction of the translation activity upon glucose removal (Fig. 7B). It would be beneficial to be able to have a CFPS system that consumes a byproduct like P_i_ as it is thought that P_i_ is main inhibiting component in CFPS [40]. Adding purified mitochondria and glutamate and malate to CFPS could consume the P_i_ and produce the ATP [71].

The use of glucose as secondary source of energy has been shown to have more positive impact on CFPS in E.coli systems more than in mammalian/eukaryotic systems, this may be attributed to the difference in glycolytic pathways between eukaryotes and prokaryotes, in prokaryotes, which lack mitochondria, glycolysis happens in the cytoplasm and more ATP is produced.

The pre-incubation experiments demonstrate that extract itself quickly consumes the ATP (Fig. 6A, 0 mM CrP point). The ATP consumption is not related to how much protein (luciferase) is synthesized *e.g.* CFPS programmed with HCV-IRES mRNA produced 30-fold lesser amount luciferase with 1 h than with capped IFITIM-mRNA, but both CFPS activities reached a plateau in an hour. We speculate that if one could specifically inhibit all the ATPases or GTPases in the extract that are not required for the CFPS then we would decrease the ATP consumption by extract and increase protein synthesis on our specific mRNA. For example, what effect would the inhibition of the V-type proton ATPase have on CFPS that are potential ATP consumers, present in the extract? In the RRL system a significant amount of free energy appears to be directed toward protein synthesis (at most 40%), but a majority supports alternative pathways that are associated with the ribosomal fraction [45].

It is thought that during CFPS byproducts start to accumulate and eventually inhibit the assay [40, 43]. These byproducts can be reduced by using a dialysis system. The main byproducts are thought to be creatine and Pi or polyphosphates. At the same time, we demonstrate that increasing the creatine phosphate concentration alone in the CFPS reaction significantly (over 5-fold) enhances protein synthesis activity. The dialysis approach further increases the duration of CFPS, although it is challenging to differentiate whether this increase comes from the reduction of byproducts or increased availability of consumable components (amino acids, ATP/GTP, CrP). Replacing the entire dialysis buffer with a fresh one did not restore the already inhibited CFPS. This suggests that certain translation factors, tRNA-synthetases, or mRNA, among others, become inhibited or degraded. The stability of different factors required for CFPS during prolonged incubation is unknown. During dialysis, we also observed the formation of a precipitate inside the dialysis membrane, which may induce the depletion of some factors. Compared to other previously published yeast or mammalian CFPS systems [34, 40, 43], our HEK293 based system outperforms 6 to 250-fold.

We are using purified recombinant GADD34 and K3L proteins that we add to our CFPS assay. A HEK293T cell line expressing these two proteins was developed just recently and is a useful addition to the CFPS approach [34]. These proteins significantly enhance the effectiveness of CFPS-based systems. However, if one wishes to study different types of cell lines or tissues, preparing an endogenously expressing cell line can be time-consuming and sometimes impossible. In such cases, a simpler approach is to add these two proteins directly to the CFPS assay.

Beside the highly regulated translation initiation the eukaryotic translation elongation is also regulated by different modifications of translation elongation factors. One such checkpoints is the phosphorylation of eEF2. When cells are starved of nutrients, eEF2 is phosphorylated by the Ca^2+^-activated kinase eEF2K, resulting in a lower binding affinity for the ribosome [58]. The activity of eEF2K is regulated by nutrients through the mTORC1 and AMPK pathway [72, 73]. Despite the major role of eEF2’s phosphorylation in blocking the bulk protein translation, its phosphorylation in neurons is associated with elevated translation of Arc/Arg3.1 which plays a key role in postsynaptic endocytosis [74]. We tested three different small-molecule inhibitors (NH125, A-484954, and AT13148) that influence eEF2 phosphorylation. We did not observe any significant effect of these inhibitors on CFPS. This may indicate that eEF2 is not inhibited through T56 phosphorylation during the measured timescale of CFPS. As we did not directly measure the eEF2 phosphorylation level during CFPS we cannot rule out that other kinases phosphorylate eEF2. For example, AMPK was implicated in direct phosphorylation of eEF2 independent of eEF2K [75, 76]. Alternatively, the pathways inhibited by those inhibitors in live cells may be inactive in the CFPS system, *e.g.* NH125 induce eEF2 phosphorylation is mediated through multiple pathways [60].

## Supplemental Materials

Supplementary data for this paper are available on-line only at http://jmb.or.kr.
