# Supplementary material for: Optimizing Human Cell-Free System for Efficient Protein Production
Source: J Microbiol Biotechnol. 2025 Feb 25;35:e2410026. doi: 10.4014/jmb.2410.10026 (PMC11896798; doi:10.4014/jmb.2410.10026)
Supplement: Supplementary file 1 [file jmb-35-e2410026-supple.pdf]

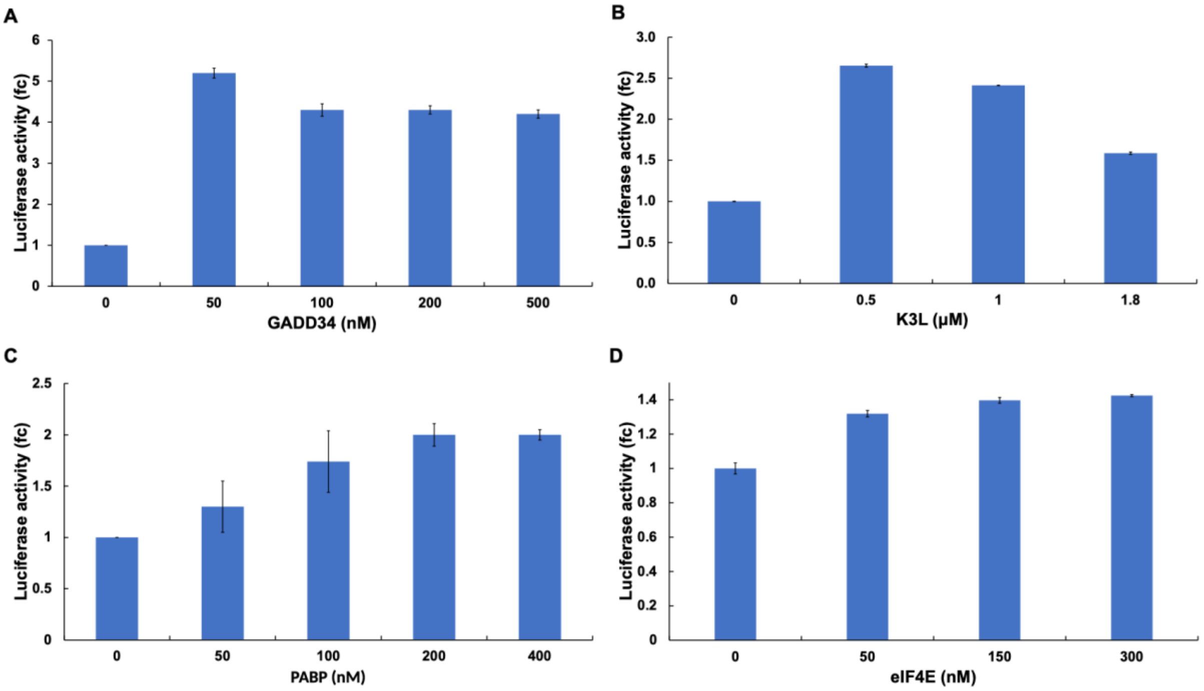

Supplementary_Figure_S1

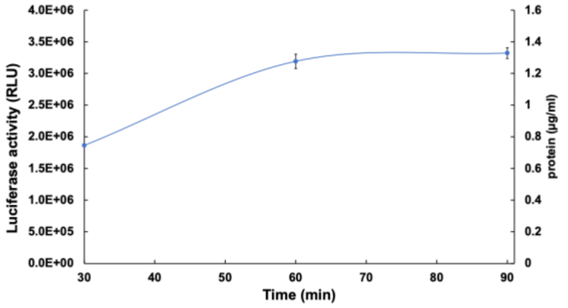

Supplementary_Figure_S2

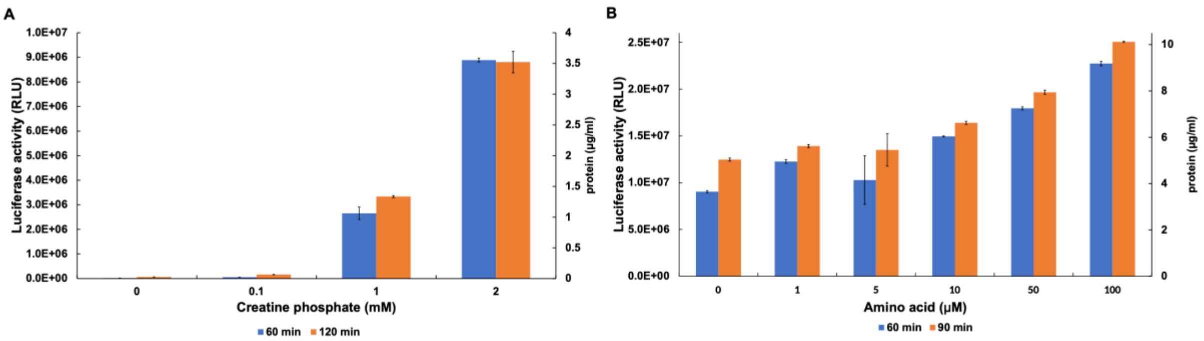

Supplementary_Figure_S3

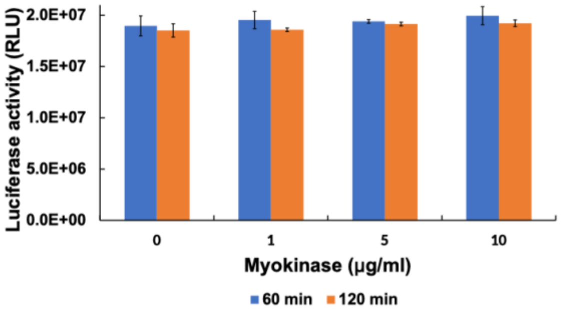

Supplementary_Figure_S4

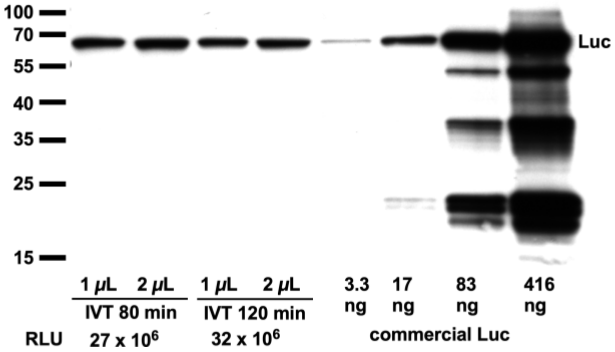

Supplementary_Figure_S5

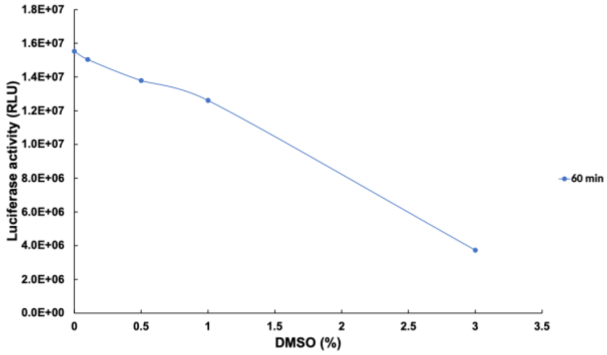

Supplementary_Figure_S6

	Fold change 90 or 120* min/60 min	Fold change 180 min/ 120 min	Fold change high/low 60 min	Fold change high/low 90 min	Fold change high/low 120 min	CrP conc. (mM)
**mRNAs**						
Cap-IFITM1-Luc	1.03*	-	-	-	-	2
EMCV	1.17*	-	-	-	-	2
CrPV	1.05*	-	-	-	-	2
HCV	1.15*	-	-	-	-	2
**Cell lines and fractions**						
Pellet1	1.04*	-	-	-	-	2
SH-SY5Y	1.04	-	-	-	-	2
**Compounds**						

**Creatine kinase exogenous (eCK)**						
eCK (0 µg/µL)	1.02*	-	1.00	-	1.00	2
eCK (0.05 µg/µL)	1.05*	-	0.98	-	1.00	2
eCK (0.25 µg/µL)	1.01*	-	0.97	-	0.97	2
eCK (0.5 µg/µL)	1.05*	-	0.95	-	0.97	2
**Creatine phosphate (CrP)**						
CrP (0 mM)	1.54*	-	1.00	1.00	1.00	
CrP (2 mM)	1.12*	-	17.01	13.71	12.39	
CrP (4 mM)	1.05*	-	35.49	27.85	25.15	
CrP (8 mM)	1.33*	-	59.40	53.45	51.44	
CrP (20 mM)	1.65*	-	56.88	55.46	60.95	
CrP (0 mM)- w/o ATP	3.97*	-	1.00	-	1.00	
CrP (0.1 mM)- w/o ATP	3.09*	-	3.16	-	2.45	
CrP (1 mM)- w/o ATP	1.26*	-	160.22	-	50.68	
CrP (2 mM)- w/o ATP	0.99*	-	536.45	-	133.80	
**Amino acids (aa)**						
aa (0 µM)	1.38	-	1.00	1.00	-	2
aa (1 µM)	1.13	-	1.36	1.12	-	2
aa (5 µM)	1.31	-	1.14	1.08	-	2
aa (10 µM)	1.1	-	1.65	1.32	-	2
aa (50 µM)	1.1	-	1.99	1.58	-	2
aa (100 µM)	1.1	-	2.52	2.01	-	2
aa (10 µM)	-	1.08	-	-	1.00	15
aa (50 µM)	-	1.03	-	-	1.38	15
aa (100 µM)	-	1.06	-	-	1.31	15
aa (145 µM)	-	1.08	-	-	1.33	15
**Creatine (Cr)**						
Cr (0 mM)	0.95	-	1.00	1.00	-	2
Cr (1 mM)	0.95	-	0.94	0.94	-	2
Cr (4 mM)	0.93	-	0.92	0.90	-	2
Cr (10 mM)	0.98	-	0.79	0.82	-	2
**Bortezomib (Br)**						
Br (0 µM)	0.96	-	1.00	1.00	-	2
Br (10 µM)	1.07	-	1.08	1.21	-	2
Br (100 µM)	1.04	-	1.23	1.34	-	2
**Spautin-1 (Sp1)**						
Sp1 (0 µM)	-	-	1.00	-	-	2
Sp1 (1 µM)	-	-	1.04	-	-	2
Sp1 (5 µM)	-	-	1.02	-	-	2
Sp1 (25 µM)	-	-	1.03	-	-	2
**NH125**						
NH125 (0 µM)	1.00*	-	1.00	-	1.00	2
NH125 (1 µM)	0.99*	-	1.00	-	0.99	2
NH125 (10 µM)	0.99*	-	1.00	-	0.99	2
NH125 (100 µM)	1.01*	-	0.96	-	0.96	2
**A-484954**						
A484954 (0 µM)	2.68*	-	1.00	-	1.00	20
A484954 (0.5µM)	2.42*	-	1.03	-	0.93	20

A484954 (5 µM)	2.33*	-	0.98	-	0.85	20
A484954 (50 µM)	2.48*	-	0.97	-	0.90	20
**AT13148**						
AT13148 (0 µM)	1.00*	-	1.00	-	1.00	2
AT13148 (1 µM)	0.94*	-	1.06	-	1.00	2
AT13148 (10 µM)	1.04*	-	0.93	-	0.96	2
AT13148(100µM)	1.07*	-	0.80	-	0.86	2
**Glucose (Glu)**						
Glu (0 mM)	1.42*	0.97	1.00	1.00	1.00	0
Glu (10 mM)	1.24*	0.90	13.21	11.49	10.63	0
Glu (20 mM)	1.20*	0.94	13.45	11.31	10.88	0
**Myokinase (Myk)**						
Myk (0 µg/ml)	0.98*	-	1.00	-	1.00	2
Myk (1 µg/ml)	0.95*	-	1.03	-	1.00	2
Myk (5 µg/ml)	0.99*	-	1.02	-	1.03	2
Myk (10 µg/ml)	0.96*	-	1.05	-	1.04	2

Supplementary Table S3.
